# Supplementary material for: Leishmania profilin interacts with actin through an unusual structural mechanism to control cytoskeletal dynamics in parasites
Source: J Biol Chem. 2024 Feb 9;300(3):105740. doi: 10.1016/j.jbc.2024.105740 (PMC10907219; doi:10.1016/j.jbc.2024.105740)
Supplement: Supporting information [file mmc1.docx]

**SUPPORTING INFORMATION**

***Leishmania* profilin interacts with actin through an unusual structural mechanism to control cytoskeletal dynamics in parasites**

**Andrea Vizcaíno-Castillo^1,^*, Tommi Kotila^1,^*, Konstantin Kogan^1,^*, Ryuji Yanase^2,^*, Juna Como^3^, Lina Antenucci^1^, Alphee Michelot^3^, Jack D. Sunter^2,#^, Pekka Lappalainen^1,#^**

**
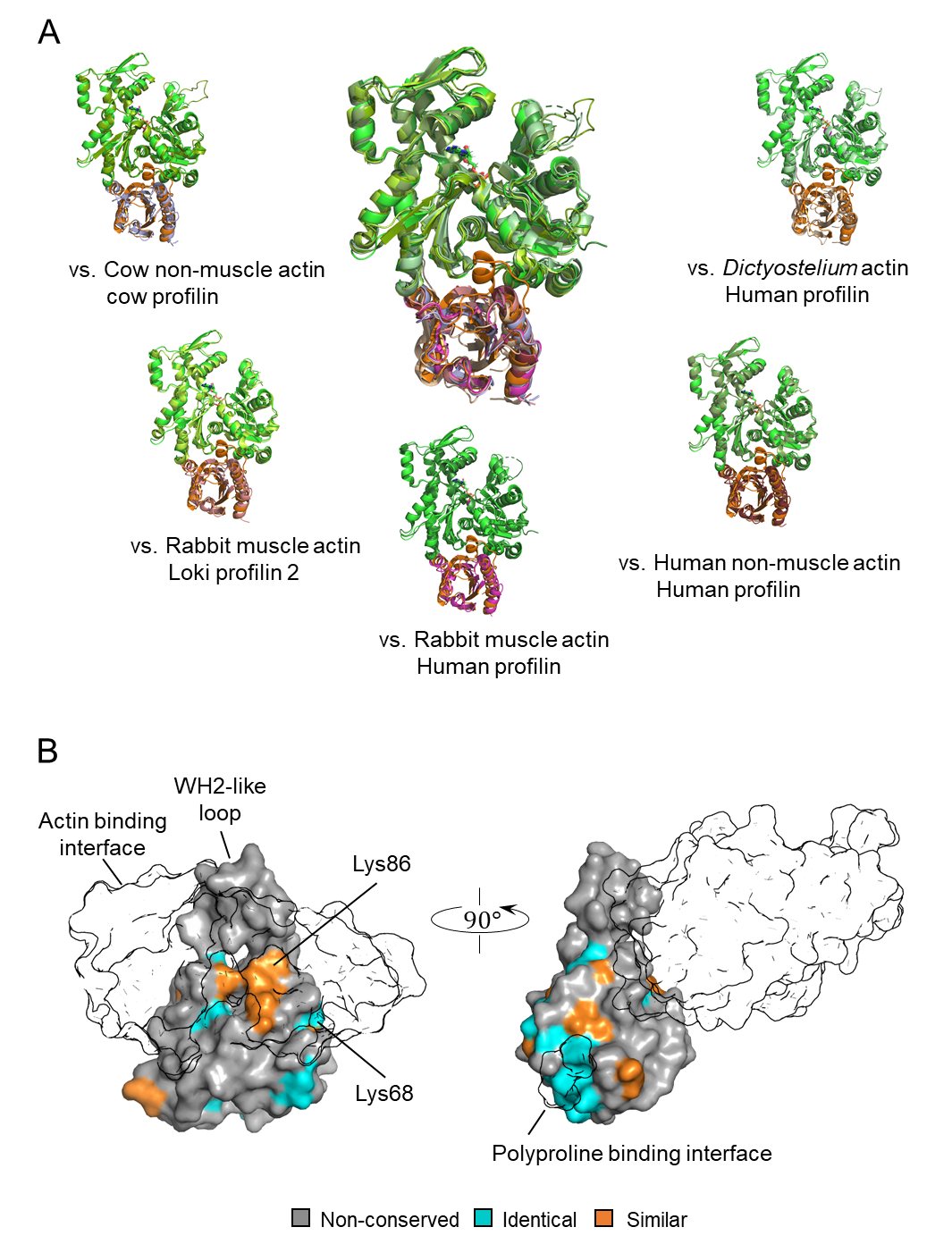
**

**Figure S1. Conservations of the structures and binding interfaces between mammalian and *Leishmania* proteins. (A)** Overlay of actin backbones from all profilin-actin co-crystal structures. **(B)** Conservation of the main actin and poly-proline binding interfaces between mammalian and *L. major* profilins. Identical and chemically similar residues are highlighted in light blue and orange, respectively, and the non-conserved residues are shown in grey. Actin and poly-proline peptide are shown as transparent outline structures.


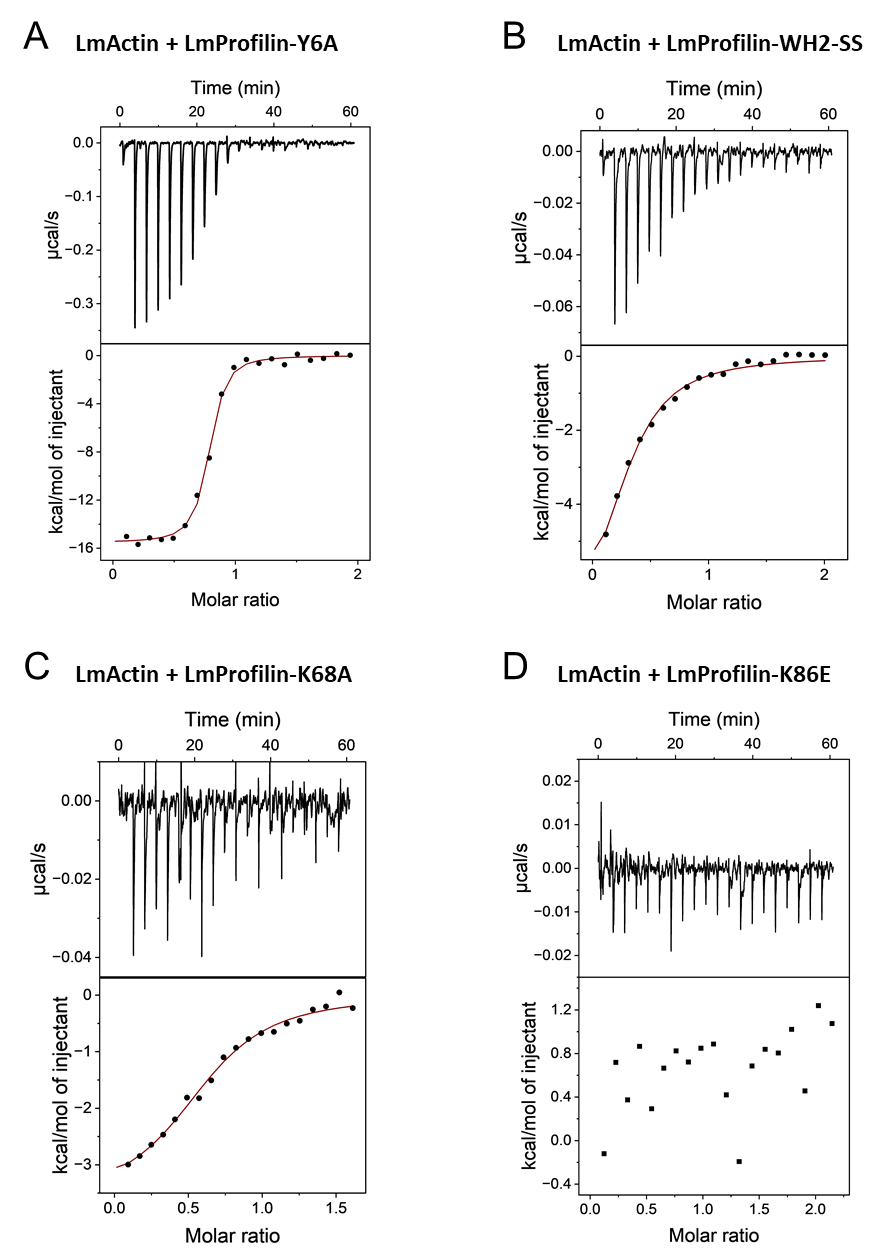


**Figure S2. ITC binding isotherms of LmProfilin mutants.** Raw (upper graphs) and integrated data fit to one-site binding model (lower graphs) of binding isotherms obtained from the ITC experiments. Representative examples from 3 independent experiments are shown for each LmProfilin titrated with LmActin. **(A)** LmProfilin-Y6A. **(B)** LmProfilin-WH2-SS. **(C)** LmProfilin-K68A. **(D)** LmProfilin-K86E.


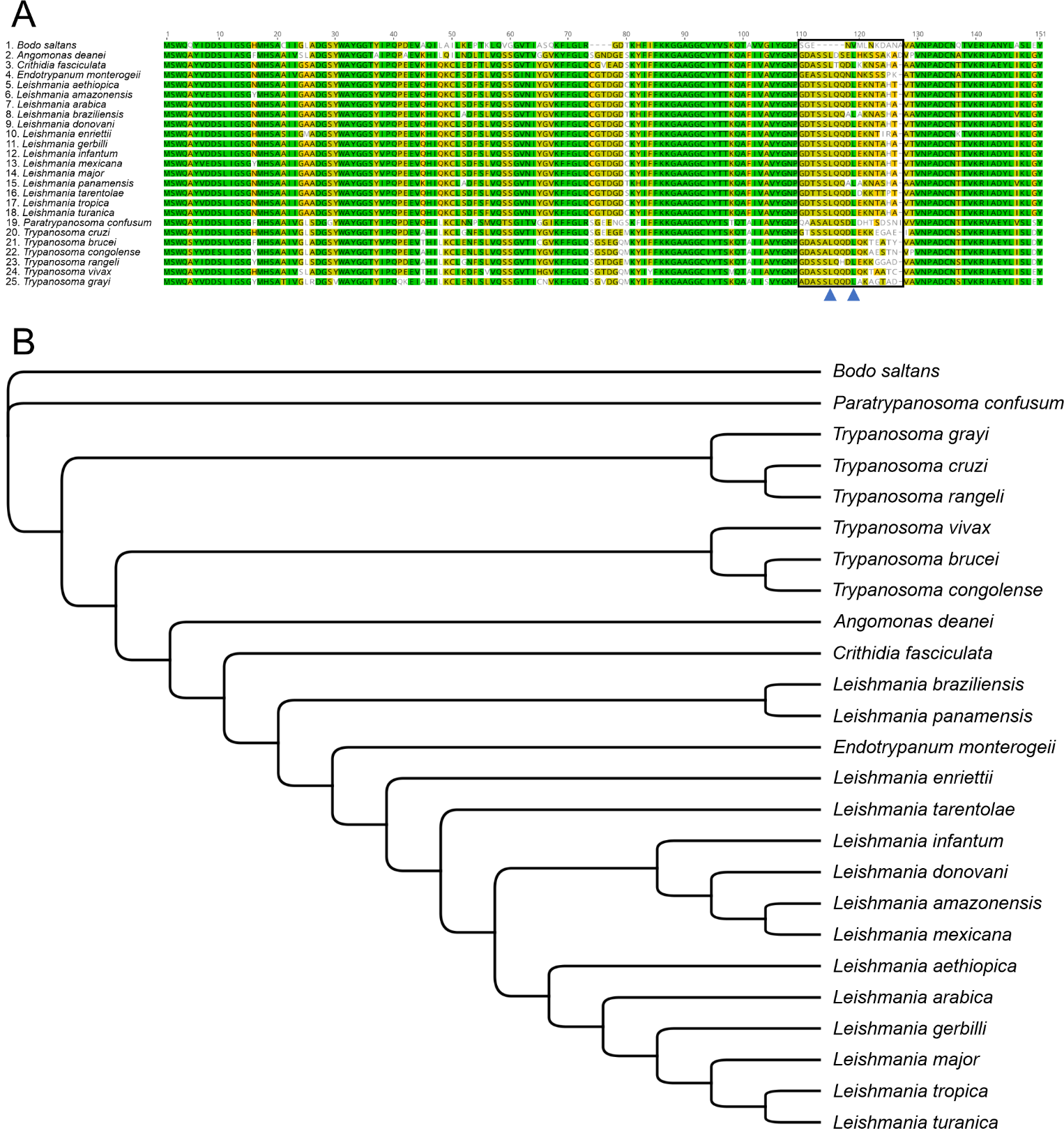


**Figure S3. The WH2 domain –like insertion is conserved across the Trypanosomatidae family. (A)** Protein sequence alignment of selected species, mainly *Leishmania* and *Trypanosoma* genera. The WH2 motif is marked by black rectangle. Two blue arrowheads highlight the two conserved hydrophobic residues, which are crucial for interactions with actin. **(B)** Phylogenetic tree based on the protein sequence alignment from panel A.

**
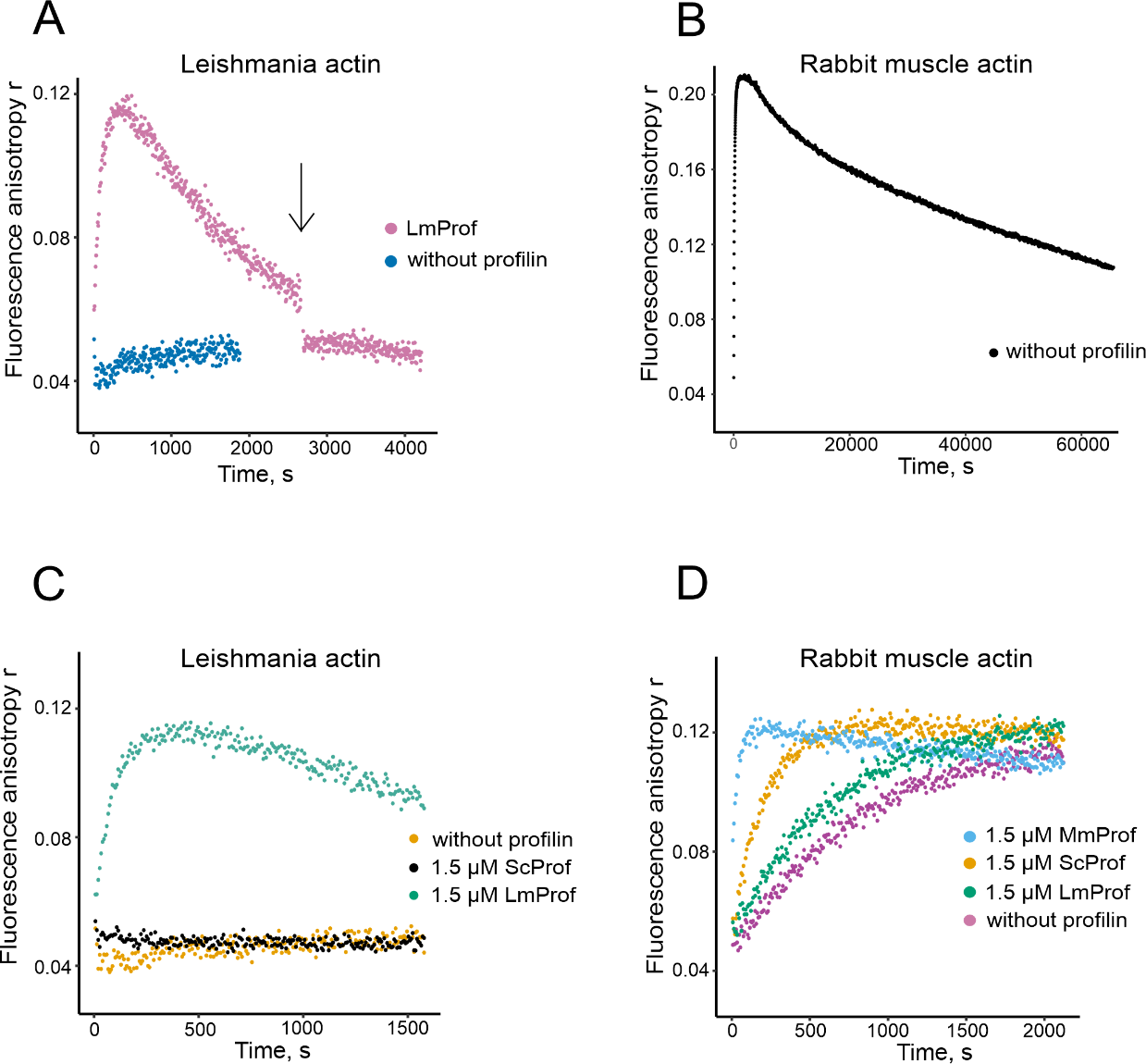
**

**Figure S4. Mammalian actin is a poor substrate for *Leishmania* profilin, and vice versa. (A)** Fluorescence anisotropy experiment with two consecutive injections of ATP-ATTO-488 (0.1 μM) at 0 s and 2658 s (black arrow). The absence of a new peak after the second injection suggests progressive inactivation or unfolding of nucleotide-free monomeric Mg-actin rather than progressive hydrolysis of ATP-ATTO-488. **(B)** Fluorescence anisotropy experiment with rabbit muscle actin and ATP-ATTO-488 (0.067 µM) showing a similar decay of the anisotropy signal for rabbit muscle actin, but on much longer time scales. **(C)** ATP-ATTO-488 (0.1 µM) fluorescence anisotropy experiment to compare the efficiency of two profilins (*L. major* or *S. cerevisiae* Pfy1p) to exchange the nucleotide bound to LmActin monomers. **(D)** ATP-ATTO-488 (0.1 µM) fluorescence anisotropy experiment to compare the efficiency of three profilins (*L. major* profilin, *S. cerevisiae* Pfy1p, and *M. musculus* PFY1) to exchange the nucleotide bound to rabbit muscle actin monomers. The data are representative of at least two independent experiments with similar results.

**
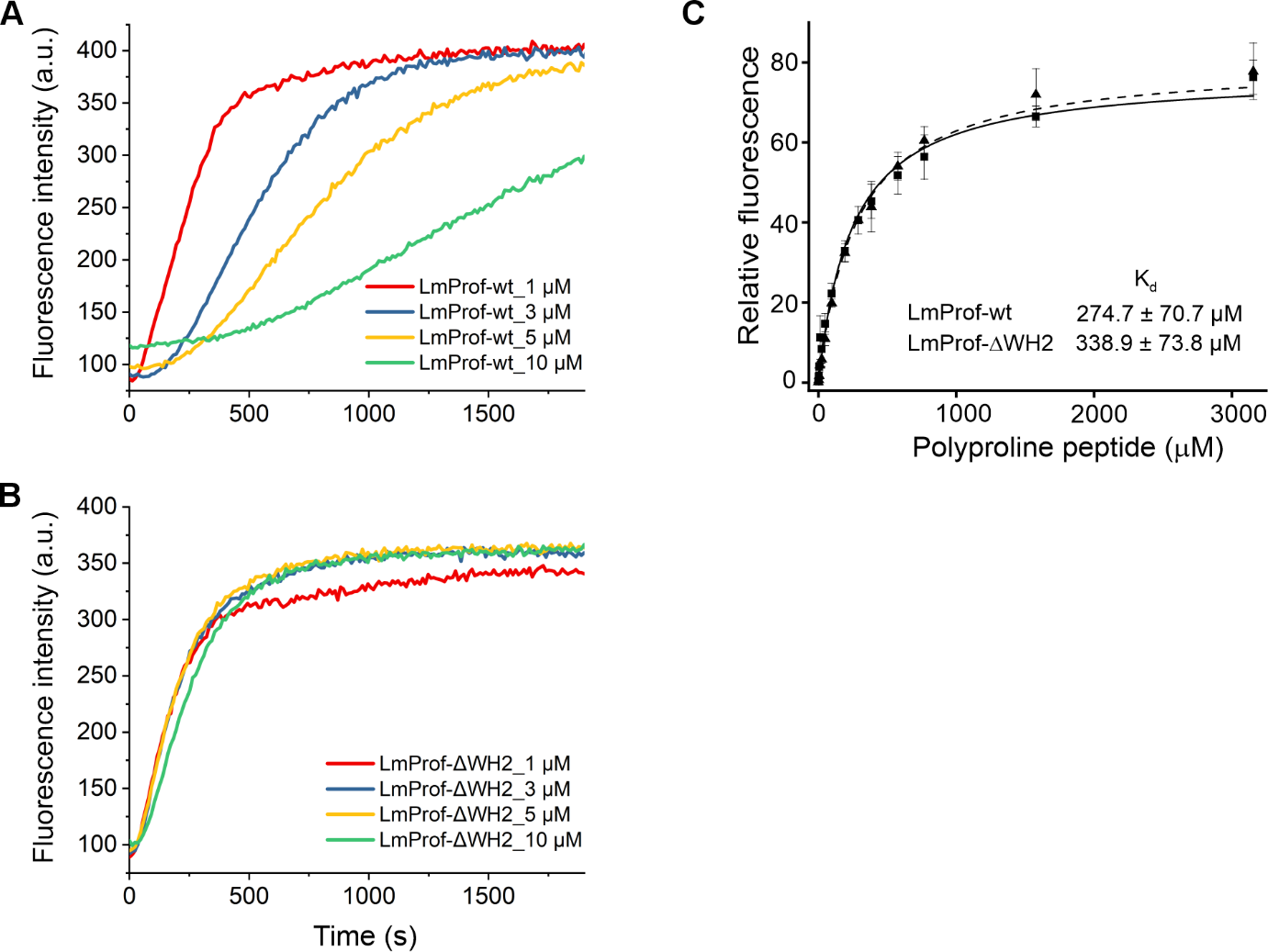
**

**Figure S5. Formin-profilin titration.** Pyrene-actin polymerization assays to evaluate the effect of different concentrations (1, 3, 5, 10 µM) of LmProfilin-wt **(A)** and LmProfilin-ΔWH2 **(B)** on LmActin polymerization in the presence of LmFormin FH1-FH2 fragment. Final concentration of actin (95% LmActin, 5% rabbit-pyrene actin) and LmFormin FH1-FH2 were 3 µM and 0.05 µM, respectively. One representative experiment of two independent assays is shown. **(C)** Tryptophan fluorescence assay to evaluate the interaction of wild-type LmProfilin (LmProf-WT; solid circles, solid line) and LmProfilin-ΔWH2 mutant (LmProf- ΔWH2; solid triangles, dashed line) with a poly-L-proline peptide. Different concentrations of a decamer poly-L-proline peptide were mixed with 1 µM of profilins and the relative fluorescent intensity was measured. Data points (mean ± S.D. from three independent experiments) are shown as symbols, and the fitting curves as lines. The obtained Kd values (mean ± S.D. from three independent experiments) for the wild-type and ΔWH2 mutant profilin - poly-L-proline interactions are shown.

**
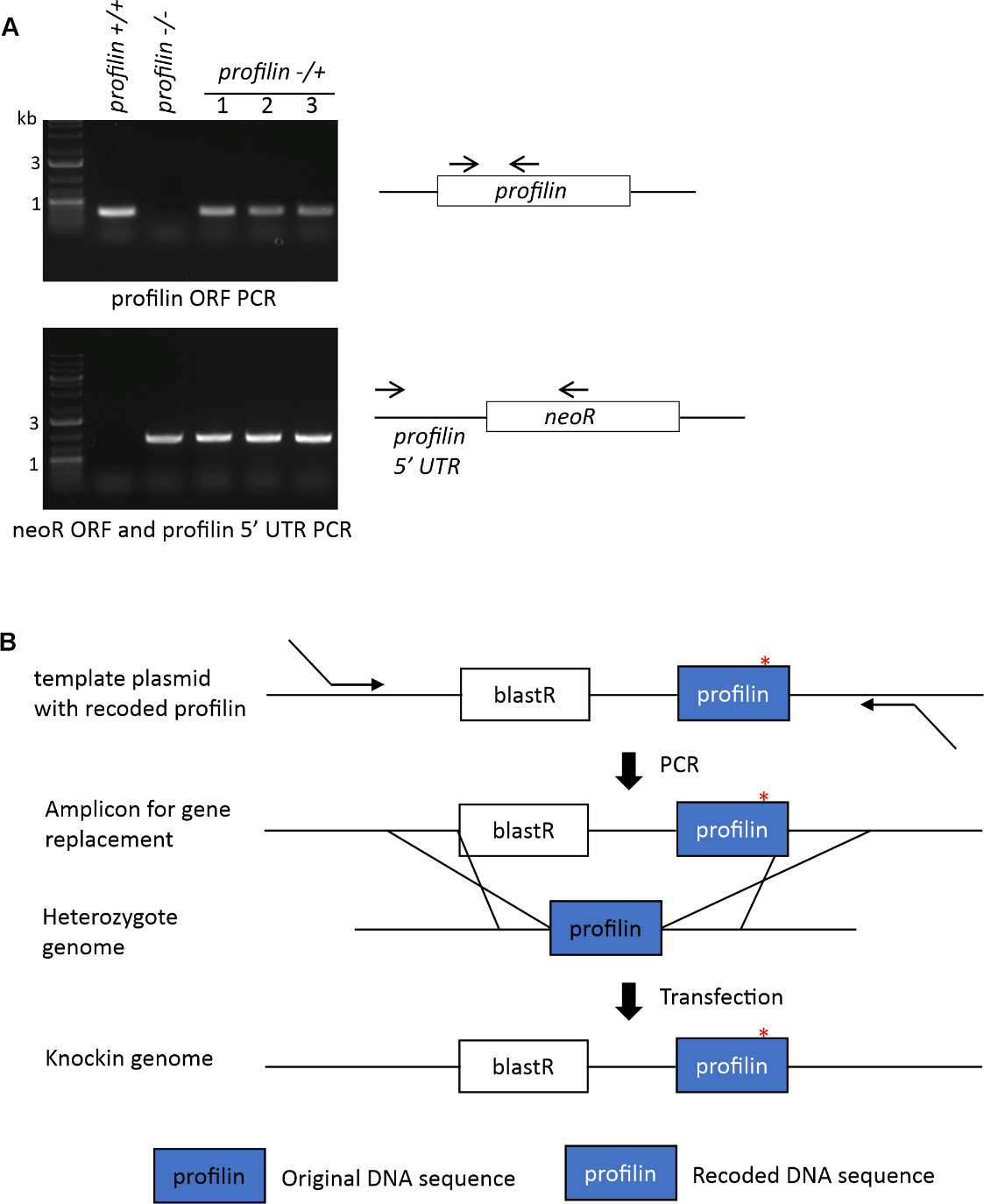
**

**Figure S6. Generation of profilin deletion and heterozygote cell lines.** **(A)** CRISPR/Cas9 mediated genome editing was used to delete one allele of the profilin gene in the parental C9/T7 cells (profilin +/+). Deletion of one allele of profilin was confirmed by PCR to check for integration of the neomycin resistance gene into the profilin locus. Three profilin heterozygotes were readily generated with the neomycin resistance gene integrating in the expected locus. Unexpectedly, in one clone both alleles of profilin were deleted. **(B)** Schematic of mutagenesis strategy. Plasmid encoded blasticidin resistance gene and recoded profilin gene sequence used as a template for long primer PCR to generate amplicon for replacement of remaining profilin allele in the heterozygote cell line. Red asterisk represents mutation introduced into the profilin gene.

**
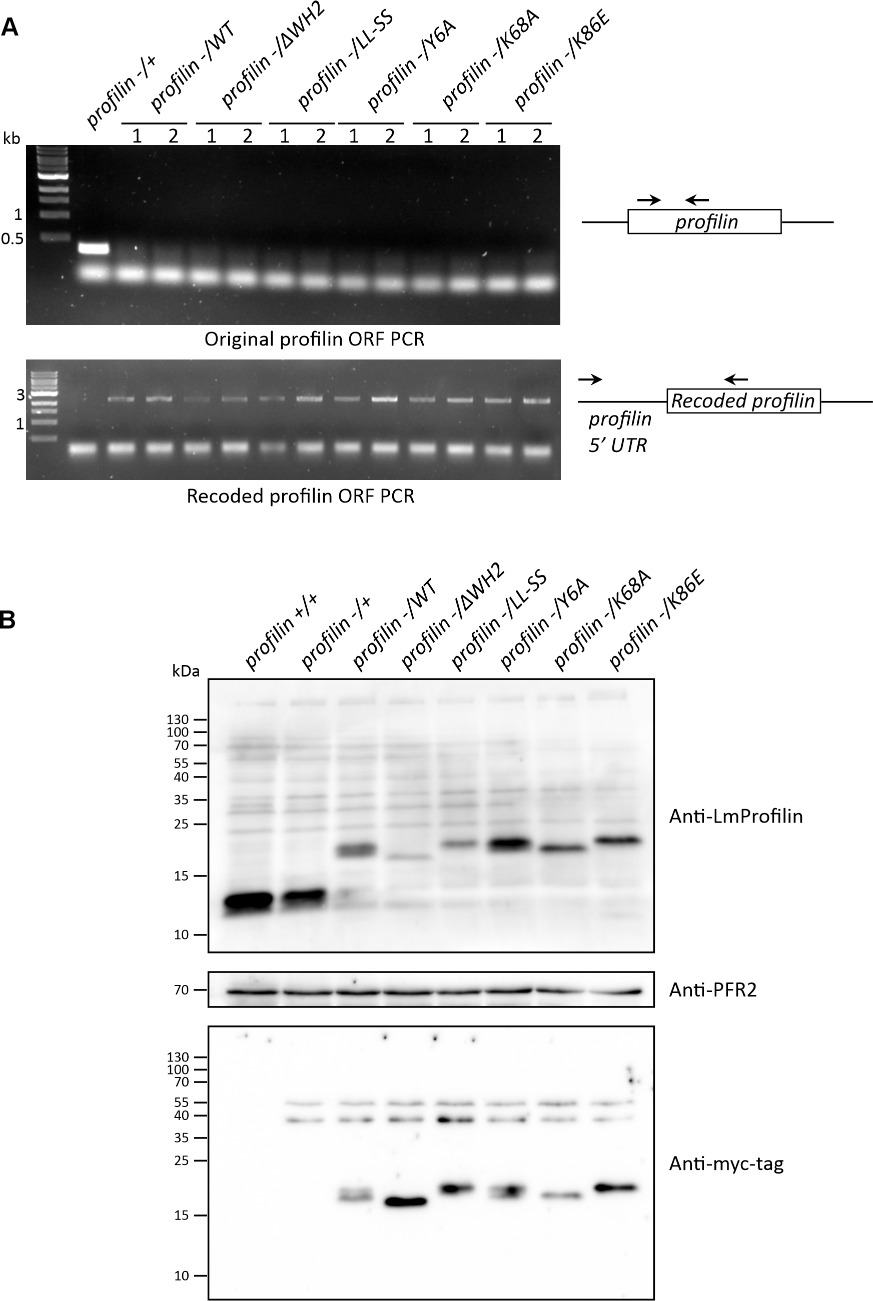
**

**Figure S7. Confirmation of profilin mutations. (A)** Deletion of original profilin allele was confirmed by PCR, with integration of mutant profilin gene into the expected locus also confirmed by PCR. Profilin ORF was sequenced to confirm that expected mutation was present. **(B)** Western blot of whole cell lysates of wild-type and mutant *L. mexicana*. Profilin was probed with anti-LmProfilin guinea pig antibody (1:500) and horseradish peroxidase-conjugated anti-guinea pig secondary antibody (1:2500) (upper blot). The membrane probed with anti-LmProfilin antibody was washed thoroughly with TBST, blocked again and a *Leishmania* paraflagellar rod protein, PFR2, was probed as a loading control with anti-PFR2 mouse antibody (L8C4, 1:1000) and horseradish peroxidase-conjugated anti-mouse secondary antibody (1:2500) (middle blot). Western blot of whole cell lysates of wild-type and mutant *L. mexicana* were also probed with anti-Myc antibody (bottom blot).

**
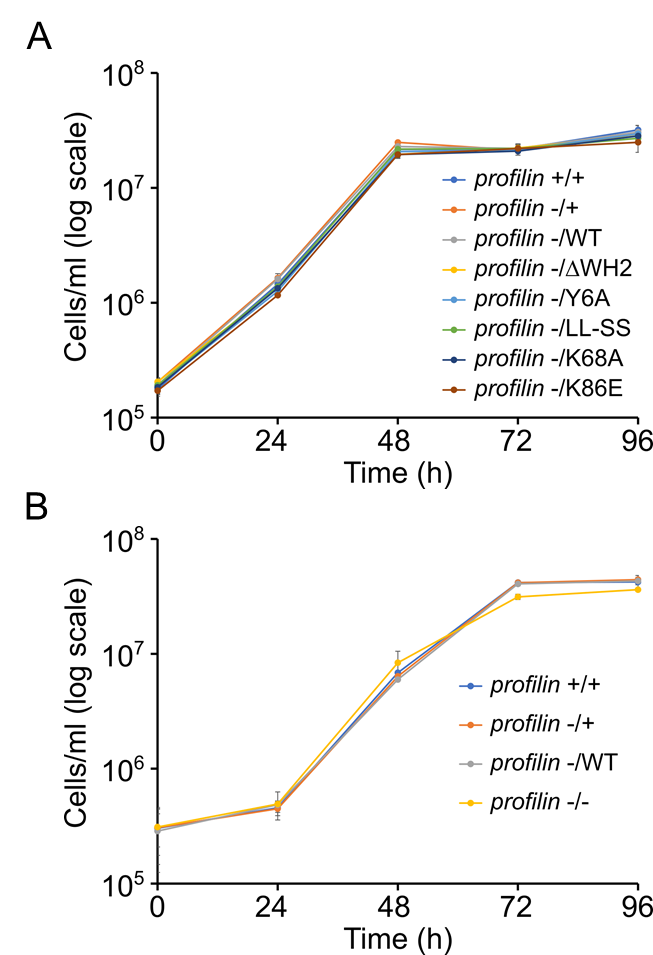
**

**Figure S8. Growth curve of profilin wild-type and mutant *L. mexicana*.** **(A)** Growth curves of profilin wild-type and mutant *L. mexicana* strains*.* **(B)** Growth curves of profilin wild-type and null mutant *L. mexicana.* Data represent mean ± SD (n = 3 independent experiments).

**Table S1. Data collection and refinement statistics.**

| ***Data collection*** | ***LmActin-Profilin*** |
| --- | --- |
| **Space group** | I 41 |
| **Cell dimensions** |  |
| a,b,c (Å) | 171.297 171.297 43.694 |
| α,β,γ (°) | 90 90 90 |
| **Resolution** | 33.59 - 2.2 Å (2.28 - 2.2 Å) |
| **Number of total reflections** | 65096 (6405) |
| **Number of unique reflections** | 32691 (3219) |
| **Multiplicity** | 2.0 (2.0) |
| **Completeness (%)** | 99.84 (99.72) |
| **R_merge_** | 0.02662 (0.3158) |
| **R_meas_** | 0.03764 (0.4466) |
| **R_pim_** | 0.02662 (0.3158) |
| **I/σI** | 12.64 (1.84) |
| **CC_1/2_** | 0.999 (0.814) |
|  |  |
| ***Refinement*** |  |
| **R_work_/R_free_** | 0.1958/0.2308 (0.3346/0.3595) |
| **Protein residues** | 509 |
| **No. atoms** |  |
| Protein | 3968 |
| Ligand/ion | 44 |
| Water | 243 |
| **B-factors** |  |
| Protein | 63.89 |
| Ligand/ion | 52.07 |
| Water | 58.47 |
| **R.m.s. deviations** |  |
| Bond lenghts (Å) | 0.002 |
| Bond angles (°) | 0.49 |
| **Ramachandran plot** |  |
| Outliers (%) | 0.20 |
| Allowed (%) | 2.98 |
| Favored (%) | 96.82 |
| **Clashscore** | 5.68 |

**Table S2. Plasmids used or generated in these study**

| **Plasmid** | **Description** | **Reference** |
| --- | --- | --- |
| **pPL1698** | pFastBac1-LmActin-HsThymosin-Hisx10 | Kotila et al 2022 |
| **pPL1718** | pCoofy3-LmProfilin-wt | Kotila et al 2022 |
| **pPL1762** | pCoofy3-LmProfilin-K68A | This study |
| **pPL1759** | pCoofy3-LmProfilin-dWH2 | This study |
| **pPL1760** | pCoofy3-LmProfilin-LL-SS | This study |
| **pPL1764** | pCoofy3-LmProfilin-K86E | This study |
| **pPL1766** | pCoofy3-LmProfilin-Y6A | This study |
| **pPL1795** | pPLOT-3xMyc-LmxProfilin-wt | This study |
| **pPL1796** | pPLOT-3xMyc-LmxProfilin-dWH2 | This study |
| **pPL1797** | pPLOT-3xMyc-LmxProfilin-LL-SS | This study |
| **pPL1798** | pPLOT-3xMyc-LmxProfilin-Y6A | This study |
| **pPL1799** | pPLOT-3xMyc-LmxProfilin-K68A | This study |
| **pPL1800** | pPLOT-3xMyc-LmxProfilin-K86E | This study |
| **pPL1723** | pCoofy3-LmForminB(536-1193) | This study |

**Table S3. Primers used in these study**

| **Primer name** | **Sequence** | **Used to make** |
| --- | --- | --- |
| **LmPRF_dLOOP_F** | **GGATTAACAGTCACGCCGGGGTTACCGTAAACG** | **pPL1759** |
| **LmPRF_dLOOP_R** | **TCTTCGCAGCAGGACTCGGAAAAGAATACAGCTCATGCCGTGAC** |  |
| **LmPRF_WH2_SS_F** | **TCCGAGTCCTGCTGCGAAGAGCTAGTGTCGCCGGGGTTAC** | **pPL1760** |
| **LmPRF_WH2_SS_R** | **ATCTATGGTGTGGCATTCTTTGGGCTTCAGTGCG** |  |
| **LmPRF_K68A_F** | **AAAGAATGCCACACCATAGATATTGACGCCG** | **pPL1762** |
| **LmPRF_K68A_R** | **GTGATTGTAAGTACATCTTTTTCGAGAAGGGAGCGGCGGGC** |  |
| **LmPRF_K86E_F** | **CTCGAAAAAGATGTACTTACAATCACCGTCCGTCC** | **pPL1764** |
| **LmPRF_K86E_R** | **CAGCTGTGGATGATTCTTTAATCGGATCTGGAAAC** |  |
| **LmPRF-Y6A_F** | **GATTAAAGAATCATCCACAGCTGCCTGCCAGGAAGGACC** | **pPL1766** |
| **LmPRF-Y6A_R** | **GGATTAACAGTCACGCCGGGGTTACCGTAAACG** |  |
